# Disseminated nocardiosis caused by *Nocardia farcinica* in a patient with colon cancer: A case report and literature review

**DOI:** 10.1097/MD.0000000000026682

**Published:** 2021-07-23

**Authors:** Eung Kyum Lee, Jin Kim, Dong-Hyuk Park, Chang Kyu Lee, Sun Bean Kim, Jang Wook Sohn, Young Kyung Yoon

**Affiliations:** a Division of Infectious Diseases, Department of Internal Medicine, Korea University College of Medicine, Seoul, The Republic of Korea; b Department of Surgery, Korea University Medical Center, Korea University College of Medicine, Seoul, The Republic of Korea; c Department of Neurosurgery, Korea University Medical Center, Korea University College of Medicine, Seoul, The Republic of Korea; d Department of Laboratory Medicine, Korea University College of Medicine, Seoul, The Republic of Korea.

**Keywords:** brain abscess, empyema, *Nocardia farcinica*

## Abstract

**Rationale:**

Nocardiosis is an uncommon and potentially life-threatening infection that usually affects immunocompromised hosts. No clinical guidelines have been established for managing this rare disease, and the optimal treatment modality remains unclear. *Nocardia farcinica*, a relatively infrequent pathogen of nocardiosis, causes a clinically aggressive infection. In addition to our patient data, our search of the literature for patients who presented with empyema caused by *N. farcinica* will provide fundamental information for optimal treatment modalities.

**Patient concerns:**

A 64-year-old man was diagnosed with empyema, 4 days following surgery for sigmoid colon cancer. Brain lesions were evaluated only after *N. farcinica* was isolated and identified as the causative pathogen through repeated culture tests.

**Diagnoses:**

*N. farcinica* was isolated from the pleural effusion and confirmed as the pathogen through 16S rRNA sequencing.

**Interventions:**

The patient was successfully treated with tube thoracotomy, neurosurgical evacuation, and a combination of trimethoprim/sulfamethoxazole plus imipenem. Long-term antibiotic therapy was required to prevent recurrence.

**Outcomes:**

Pyothorax showed a good clinical response to antimicrobial therapy and drainage of pleural effusion, whereas brain abscess did not respond to medical therapy and required surgery. The patient eventually recovered and continued chemotherapy as treatment for sigmoid colon cancer.

**Lessons:**

Although extremely rare, this report demonstrates the importance of considering *Nocardia* infection as the differential diagnosis in immunocompromised patients who present with empyema. In particular, because of the *N. farcinica* infection's tendency to spread and the resistance of the organism to antibiotics, aggressive evaluation of metastatic lesions and standardized support from microbiological laboratories are important. Surgery may be required in some patients with brain abscesses to improve the chance of survival.

## 1 Introduction

Nocardiosis in humans was first reported by Eppinger in 1890.^[1]^ It is an opportunistic infection caused by Gram-positive, partially acid-fast, methenamine silver-positive filamentous aerobic bacteria, which are generally found in soil, dust, and water.^[2]^ Nocardiosis can present with protean clinical syndrome, but the most common site of infection is the lung, which develops by inhalation or direct inoculation of *Nocardia* organisms.^[3–5]^ Approximately 50% of all patients with pulmonary infection demonstrate spread of disease to the extra-pulmonary sites, most commonly the brain. By contrast, 20% of extrapulmonary nocardiosis infections occur without pulmonary disease.^[1]^

At least 219 species have been described to date with the rapid growth of cases caused by the genus *Nocardia*.^[6]^*Nocardia asteroids*, which are included in the *N. asteroides* complex, were formerly considered the most common species associated with human infections.^[7]^ However, the distribution and susceptibility profiles available worldwide demonstrate geographical variations and changes over time.^[3,4,8,9]^*Nocardia farcinica* is one of the less common species among the various species. It is more virulent and resistant to antibiotics than *N. asteroids,* especially third-generation cephalosporins and tobramycin.^[10–12]^ Eventually, it is associated with a high risk of disseminated infection and mortality.^[3,9,10]^

*Nocardia* species are not considered normal commensal organisms.^[13]^ Rarely, *Nocardia* species may be found as contaminants in the skin and upper respiratory tract. However, the isolation of *Nocardia* from respiratory specimens should be carefully evaluated for true infection accompanied by disseminated disease, particularly in immunocompromised hosts.^[1]^*Nocardia* species not only grow poorly in some culture media, but may also take more than 5 days to grow. Therefore, multiple specimens may be required for culturing for a sufficient time.

Although antimicrobial susceptibility differs according to the various species of *Nocardia* isolates, there is still no standardized method for antimicrobial susceptibility testing to guide clinical treatment. In addition, there is extremely limited information available for physicians to diagnose nocardiosis early or for selecting the appropriate antibiotic therapy. Herein, we describe an immunosuppressed patient diagnosed with disseminated nocardiosis caused by *N. farcinica*. Furthermore, a systematic review of the literature on nocardiosis manifesting as empyema due to *N. farcinica* was performed to identify the main strategies for improving the prognosis of these patients.

## 2 Case report

**Figure 1:**
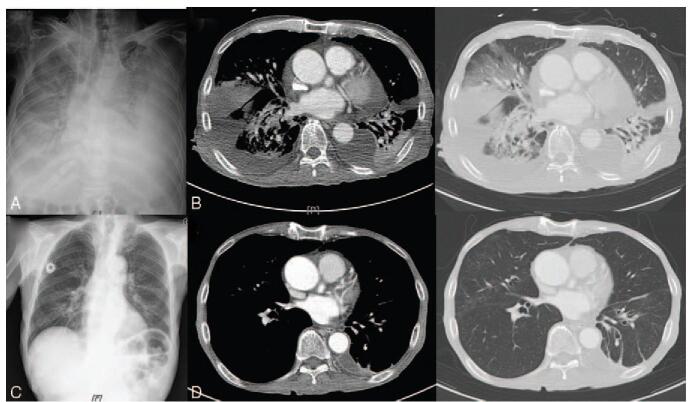
(A) Chest radiography demonstrated progressive patchy consolidations in the bilateral lung fields and bilateral pleural effusion. (B) Chest computed tomography (CT) scan shows encapsulated effusion surrounded by irregularly thickened pleura. (C) Chest radiography on 70th treatment day suggested improvement of patchy consolidations but remained atelectic. (D) Chest CT on 52nd treatment day revealed decreased extent of consolidations and patchy ground-glass opacity lesions in both lungs.

A 64-year-old man was diagnosed with sigmoid colon cancer (stage IIC, pT4bN0) by colonoscopy because of constipation that lasted for a month. He had no specific medical history but had smoked for 20 years. He had an ulcerating mass 15 cm above the anal verge and was diagnosed with adenocarcinoma. Although he had metastatic lung lesions, he underwent laparoscopic anterior resection as palliative surgery on the 13th day of hospitalization. Four days after the surgery, he presented with cough and dyspnea for 2 days (New York Heart Association class III). The patient's body weight was 59.8 kg, and the body mass index was 21.4 kg/m^2^. Upon physical examination, he had a body temperature of 37.1°C, respiratory rate of 20 breaths/min, pulse rate of 103 beats/min, and blood pressure of 85/55 mmHg. Pulmonary auscultation revealed crackles on both lung bases. He had no visible cutaneous lesions. Laboratory data showed the following results: a white blood cell count of 13,960/mm^3^ (neutrophil: 92.7%, lymphocyte: 4.2%, monocyte: 2.7%, and eosinophil: 0.4%), hemoglobin level of 7.9 g/dL, platelet count of 226,000/μL, C-reactive protein level of 162.12 mg/dL, procalcitonin level of 0.323 ng/mL, blood urea nitrogen level of 9.7 mg/L, creatinine level of 0.33 mg/dL (creatinine clearance: 136 mL/min), and albumin level of 1.9 g/dL. The patient's D-dimer level was 4.15 μg/mL. A chest radiograph showed multiple patchy consolidate-like shadows and massive pleural effusion in both lungs, which was more obvious in the left lung (Fig. 1A). Chest computed tomography (CT) revealed the presence of fluid encapsulated by an irregularly thickened pleural membrane (Fig. 1B).

**Figure 2:**
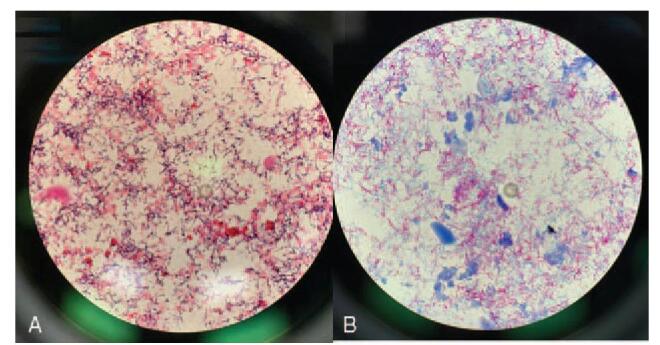
(A) Microbiological staining of the pleural fluid specimens disclosed Gram-positive branching bacilli. (B) Modified acid-fast stain showed the filamentous branching hyphae of *Nocardia farcinica* (×400).

Piperacillin/tazobactam was administered after sputum and blood cultures were collected. Chest tube was inserted for drainage of pleural effusion on the left side; analysis of the drained pleural effusion yielded the following results, which led to the diagnosis of empyema thoracis: protein, 1.8 g/dL; lactate dehydrogenase, 5994 IU/L; glucose, 10 mg/dL; adenosine deaminase, 28.9 U/mL; and white blood cell count, 35,800/mm^3^. Patient's serum protein and lactate dehydrogenase levels were 3.9 g/dL and 874 IU/L, respectively. His clinical condition gradually deteriorated; microbiological staining of the pleural fluid specimens revealed gram-positive branching bacilli (Fig. 2A), while modified acid-fast staining showed acid-fast bacilli (Fig. 2B). Multiple specimens were collected from the patient: blood (n = 5), sputum (n = 2), and pleural effusion (n = 4). Sputum and peripheral blood cultures were negative after incubation for 5 days. Cytology analysis of the pleural fluid showed absence of malignant cells. After 5 days of incubation, a chalky white-colored colony developed on sheep blood agar culture of only one of the pleural fluid specimens (Fig. 2C). *N. farcinica* was identified using matrix-assisted laser desorption ionization-time of flight (MALDI-TOF) mass spectrometry (Bruker Daltonics, Bremen, Germany). The GenBank Basic Local Alignment Search Tool was searched, which revealed that the 16S rRNA gene sequence of the isolate showed 99% homology of 1,458 base pairs to the corresponding sequences of the previously sequenced *N. farcinica* (GenBank accession number: LN868938.1). Antimicrobial susceptibility testing using the disk diffusion method, showed susceptibility to trimethoprim/sulfamethoxazole (37 mm), imipenem (36 mm), and amikacin (27 mm), but resistance to ciprofloxacin, vancomycin, penicillin, gentamicin, and tetracycline.

**Figure 3:**
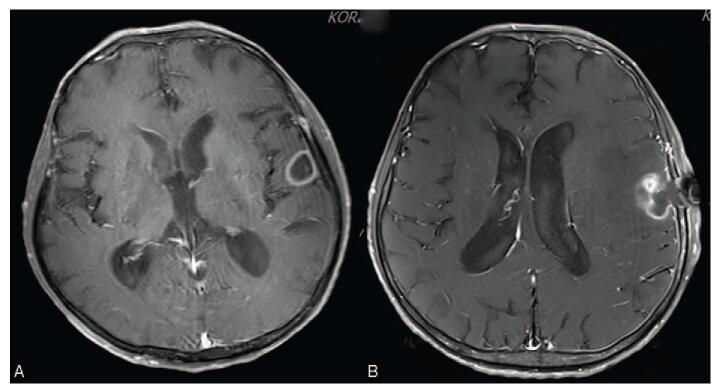
(A) A brain MRI showed rim-enhancing quasi-circular space-occupying lesion on the left frontotemporal lobes, suggesting brain abscess. (B) A brain MRI after the neurosurgery revealed deformed rim-enhancing lesion with perilesional edema.

On the 22nd day of hospitalization, initial empirical antibiotic therapy with intravenous trimethoprim/sulfamethoxazole (15 mg/kg in 4 divided doses) and imipenem (500 mg every 6 hours) was initiated as treatment for thoracic empyema. The chest tube was removed on the 22nd day after admission. On the 30th day of hospitalization, dysphagia and dysarthria became apparent as the shortness of breath improved. Brain magnetic resonance imaging (MRI) demonstrated a rim-enhancing 24-mm-diameter nodule on the left frontotemporal lobes, suggesting a brain abscess (Fig. 3A). Because general anesthesia and neurosurgery were not permitted due to patient's poor general condition, medical therapy was administered as maintenance treatment.

On the 50th day of hospitalization, a follow-up brain CT scan revealed an increase in rim-enhancing lesions and the extent of peri-lesional edema. Since the patient's condition was restored and he eventually agreed to undergo brain surgery, stereotactic abscess removal was performed. *N. farcinica* could not be isolated from intraoperative pus culture. After neurosurgical operation, patient's dysphagia, dysarthria, and general condition have eventually improved. After a 7-week course of parenteral antibiotic treatment with trimethoprim/sulfamethoxazole and imipenem without developing any adverse events, he was eventually discharged and was prescribed with oral trimethoprim/sulfamethoxazole (80/160 mg q 12 h). He has been on oral antibiotics for 4 months, and no recurrence was detected in the follow-up imaging performed 3 months after discharge (Figs. 1C, D and 3B); the patient is currently undergoing outpatient chemotherapy for sigmoid colon cancer.

## 3 Literature review

The demographic and clinical characteristics of the patients who presented with nocardial empyema caused by *N. farcinica* reported in the literature are summarized in Table 1.^[14–22]^ A total of 11 patients, including our patient, were identified. Of them, 10 (90.9%) were men with a mean age (± standard deviation) of 60.5 ± 18.3 years, ranging from 27 to 82 years. Six patients (54.5%) were aged 65 years and older, and 80% (4/5) aged 65 years and below were immunocompromised. Eight patients (72.7%) had significant underlying diseases, as listed in Table 1. Disseminated nocardiosis was reported in 6 patients (54.5%). In addition to empyema, various organs were involved, including the brain (n = 4), thyroid gland (n = 1), knee (n = 1), spine (n = 2), eye (n = 1), and skin (n = 1). *N. farcinica* isolated from 5 patients with antimicrobial susceptibility data were all susceptible to trimethoprim-sulfamethoxazole (TMP-SMX), and some showed susceptibility to imipenem, fluoroquinolones, and amikacin. The most commonly administered antibiotics were TMP-SMX (n = 6), followed by imipenem (n = 4), minocycline (n = 3), amikacin (n = 2), ciprofloxacin (n = 1), and moxifloxacin (n = 1). Except for 2 patients without relevant data, all 9 patients needed surgical intervention, while 2 (22.2%) died. The median duration (range) of antibiotic therapy was 180 days (range, 2–730 days).

**Table 1:** Clinical characteristics of patients with empyema caused by *Nocardia farcinica* in the literature.

Case no.	Reference	Age/sex	Type of infection	Underlying diseases	Medication history	Clinical presentation	Site of infection	Antimicrobial susceptibility	Surgical treatment	Antimicrobial treatment (total duration, days)	Outcome
1	Ishiguro et al^[14]^	82/M	Community acquired	Pneumoconiosis and diabetes mellitus		Chest pain, dyspnea, and knee joint pain	Lung and right knee	TMP-SMX (S), minocycline (S), levofloxacin (S), and amoxicillin/CA (S)	Yes; lung (thoracostomy) and knee (aspiration)	Minocycline and imipenem (180)	Survival
2	Graat et al^[15]^	54/M	Community acquired	Chronic liver disease and hypertension		Fever and low back pain	Lung, spinal osteomyelitis, brain abscess, and psoas muscle	–	Yes; psoas muscle and epidura (abscess decompression) L1–2 (corpectomy)	Minocycline, imipenem, ciprofloxacin, and sulfadiazine (730)	Survival
3	Chansirikarnjana et al^[16]^	69/M	Healthcare associated	Multiple myeloma	Chemotherapy (bortezomib, lenalidomide, and dexamethasone)	Fever, dyspnea, and muscle weakness	Lung, brain abscesses, and mycotic aneurysm	TMP-SMX (S) and moxifloxacin (S)	Yes; lung (thoracostomy) and brain (aneurysm clipping)	TMP-SMX and moxifloxacin (360)	Survival
4	Ando et al^[17]^	69/F	Community acquired	Idiopathic thrombocytopenic purpura	Methylprednisolone and danazol	Dyspnea	Lung	–	Yes; lung (thoracostomy)	TMP-SMX and imipenem, minocycline (365)	Survival
5	Parande et al^[18]^	27/M	Community acquired	Human immunodeficiency virus		Cough	Lung	TMP-SMX (S), amikacin (S), imipenem (S), ciprofloxacin (S), linezolid (S), and amoxicillin/CA (S)	Yes; lung (thoracostomy)	Amikacin and TMP-SMX (180)	Death
6	Tsukamura and Ohta^[19]^	70/M	Community acquired	None	–	Fever, cough, and sputum	Lung	–	Yes; lung (aspiration)	Sulfixomezole (−)	Survival
7	Severo et al^[20]^	75/M	Community acquired	Chronic low-back pain	Methylprednisolone	Fever and sputum	Lung and thyroid	–	Yes; lung (aspiration)	TMP-SMX (2)	Death
8	Özen et al^[21]^	24/M	Community acquired	Systemic lupus erythematosus	Methylprednisolone, mycophenolate mofetil	Fever and low back pain	Lung, spine, eye, brain, and skin	TMP-SMX (S), linezolid (S), imipenem (S), and ciprofloxacin (S)	Yes; paravertebral abscess (aspiration)	TMP-SMX, amikacin (42)	Survival
9	Huang et al^[22]^	56/M	–	None	–	–	Lung	–	–	–	–
10	Huang et al^[22]^	76/M	–	None	–	–	Lung	–	–	–	–
11	Present case	64/M	Healthcare associated	Colon cancer		Dyspnea	Lung and brain abscess	TMP-SMX (S), amikacin (S), and imipenem (S)	Yes; lung (thoracostomy) and brain (drainage)	TMP-SMX and imipenem (≥ 95, on medication)	Survival

F = female, M = male, TMP-SMX = trimethoprim-sulfamethoxazole.

## 4 Discussion

Our study presented the data on a rare cases of a patient who developed empyema and brain abscess caused by *N. farcinica* as a nosocomial infection. This is the first case report of empyema caused by *N. farcinica* that occurred sporadically in the absence of epidemiologic data on nocardiosis in the Republic of Korea. Data found in the literature review suggests that empyema caused by *N. farcinica* can often occur as a diverse form of disseminated disease, and that thorough microbiological examinations and optimal surgical intervention are critical for improving the outcome.

In recent years, nocardiosis has been increasingly reported worldwide.^[23]^ The distribution of most species remains stable over time,^[23]^ but *N. farcinica* is exceptionally increasing in proportion depending on the region.^[24]^ To the best of our knowledge, cases of disseminated infection with empyema caused by *N. farcinica* have not been reported to date in the Republic of Korea. However, various types of localized infections caused by *N. farcinica* have often been reported, including catheter-related bloodstream infection,^[25]^ brain abscess,^[26]^ bursitis,^[27]^ mediastinitis,^[28]^ and soft tissue infections.^[29]^ Although *Nocardia* species can infect immunocompetent hosts,^[30]^ they are generally recognized as important opportunistic pathogens in immunocompromised hosts.^[31]^ As shown in our case, an association between malignancy and nocardiosis has been well recognized.^[32]^

A relatively uncommon pathogen of nocardiosis, *N. farcinica*, has often caused clinically aggressive infection of disseminated type, especially in immunocompromised hosts. *N. farcinica* has been recognized as a human pathogen since 1975.^[33]^ Respiratory tracts and surgical wounds are considered the ports of entry in the majority of cases.^[11]^ In our patients, there was no evidence of surgical-site infections. A previous study identified *N. farcinica* as a nosocomial pathogen that caused an infection in 3 patients in the same ward over a 6-month period, but human-to-human transmission of nocardiosis is considered to be improbable.^[34]^ Our patient was confirmed to have nosocomial infection, but there was no evidence of nosocomial transmission. Considering that *Nocardia* species is not a normal flora, *Nocardia* species should be considered when implementing infection control especially among immunocompromised patients.^[35]^

A recent report suggested that central nervous system infection was present in one-third of patients with nocardiosis caused by *N. farcinica* species.^[11]^*N. farcinica* species showed a tendency to disseminate because of its virulence. Therefore, even in patients without extrapulmonary manifestations, the risk of dissemination to the brain, skin, kidney, joints, bones, or eyes should be evaluated aggressively. Disseminated nocardiosis is presumed to occur via hematogenous spread into distant organs, particularly the brain. However, as in other reports, detection of the organism in blood cultures is unusual, as in our case. A previous study suggested that recovery rates after blood cultures might be increased when the clinical suspicion of nocardiosis is communicated to the microbiological laboratory.^[36]^ To support the growth of *Nocardia* isolates, the use of selective culture media and incubation of blood cultures for a longer period should be considered.^[36]^ A previous study showed that the median time required for pathogen isolation in various samples was 4 days.

A combination of antimicrobial therapy and appropriate surgical drainage may be required to improve the outcomes in some individuals with brain abscess or empyema, as in our patient. Our patient with brain abscess did not respond to a month of antimicrobial therapy; the abscess was eventually removed through surgical drainage. In a recent report on 5 immunocompetent patients with nocardiosis, all of them underwent surgical drainage for brain abscess caused by *N. farcinica.*^[37]^

In terms of medical treatment, the most appropriate antibiotic regimens currently suggested depend on the species identified and results of antimicrobial susceptibility testing. Because each *Nocardia* species shows a specific antimicrobial susceptibility pattern, reliable microbiological data is essential for selecting the initial antibiotic therapy. In our case, the results of species identification between MALDI-TOF mass spectrometry and molecular analysis were consistent with each other, indicating the usefulness of MALDI-TOF mass spectrometry.^[38]^ A previous study demonstrated that *N. farcinica* species were relatively more resistant to multiple antimicrobial agents, including third-generation cephalosporins and aminoglycosides, except amikacin, clarithromycin, and minocycline, and susceptible to imipenem, linezolid, and amikacin.^[23]^ Notably, the resistance rate of this species to trimethoprim/sulfamethoxazole was also approximately 50%; hence, an antimicrobial susceptibility test for trimethoprim/sulfamethoxazole is required,^[22,23]^ although it has been selected as the first choice for empirical treatment in patients with nocardiosis.

In our review, the outcome was specified in 9 patients, 2 of whom died, resulting in an overall mortality rate of 22.2%. A previous study involving various forms of nocardial infections reported a mortality rate of 31%.^[11]^ It is noteworthy that patients’ immune condition, delayed diagnosis resulting from improper isolation of the pathogen, and inappropriate therapy could result in lethal outcomes.

## 5 Conclusion

Nocardiosis caused by *N. farcinica* is a disease that predominately affects immunocompromised patients. Although blood cultures rarely show positive results, the bacteria may disseminate from the primary site of infection. Due to non-specific clinical manifestations and difficulties in the identification of pathogens, nocardiosis is often mistaken for common bacterial infections. Therefore, *N. farcinica* should be considered in the differential diagnosis of empyema as an important pathogen that can develop in immunocompromised patients. Trimethoprim/sulfamethoxazole is the first-line empirical antibiotic therapy, but susceptibility testing is helpful in tailoring antibiotic regimens. Therefore, it is very important to obtain initial isolates from patients.

## Author contributions

**Conceptualization**: Young Kyung Yoon

**Data curation**: Eung Kyum Lee and Young Kyung Yoon

**Formal analysis**: Eung Kyum Lee, Chang Kyu Lee, and Young Kyung Yoon

**Funding acquisition**: Young Kyung Yoon

**Investigation**: Eung Kyum Lee, Jin Kim, Dong-Hyuk Park, and Chang Kyu Lee

**Writing – original draft**: Eung Kyum Lee

**Writing – review and editing**: Sun Bean Kim, Jang Wook Sohn, and Young Kyung Yoon
